# Characterization of Solar-Aged Porous Silicon Carbide for Concentrated Solar Power Receivers

**DOI:** 10.3390/ma14164627

**Published:** 2021-08-17

**Authors:** Inmaculada Cañadas, Victor M. Candelario, Giulia De Aloysio, Jesús Fernández, Luca Laghi, Santiago Cuesta-López, Yang Chen, T. James Marrow, Antonio Rinaldi, Ana Mariblanca Sanchez, Angelo Tatì, Claudio Testani

**Affiliations:** 1 Materials for Concentrating Solar Thermal Technologies Unit, CIEMAT-PSA Almeria, 04200 Tabernas, Spain; i.canadas@psa.es; 2 LiqTech Ceramics A/S, Industriparken 22C, 2750 Ballerup, Denmark; vcl@liqtech.com; 3 CertiMaC soc.cons. a r.l., 48018 Faenza, Italy; l.laghi@certimac.it; 4 Point-Focus Concentrating Solar Thermal Technologies Unit, CIEMAT-PSA Almeria, 04200 Tabernas, Spain; jesus.fernandez@psa.es; 5 Fundación ICAMCyL-Centro Internacional de Materiales Avanzados y Materias Primas de Castilla y León, 24009 León, Spain; director.general@icamcyl.com; 6 Department of Materials, University of Oxford, Oxford OX1 2JD, UK; yang.chen@materials.ox.ac.uk (Y.C.); james.marrow@materials.ox.ac.uk (T.J.M.); 7 Sustainability Department, ENEA, 00196 Rome, Italy; antonio.rinaldi@enea.it (A.R.); angelo.tati@enea.it (A.T.); 8 Asociación Española de Normalización, UNE, 28004 Madrid, Spain; amariblanca@une.org; 9 Consorzio CALEF C/O ENEA CR Casaccia S.M. di Galeria, 00123 Roma, Italy; claudio.testani@consorziocalef.it

**Keywords:** silicon carbide, accelerated ageing, non-destructive testing, computed tomography, ultrasonic, thermal diffusivity, thermal conductivity, concentrated solar power plants

## Abstract

Porous silicon carbide is a promising material for ceramic receivers in next-generation concentrated solar power receivers. To investigate its tolerance to thermal shock, accelerated ageing of large coupons (50 × 50 × 5 mm) was conducted in a solar furnace to investigate the effects of thermal cycling up to 1000 °C, with gradients of up to 22 °C/mm. Non-destructive characterization by computed X-ray tomography and ultrasonic inspection could detect cracking from thermal stresses, and this informed the preparation of valid specimens for thermophysical characterization. The effect of thermal ageing on transient thermal properties, as a function of temperature, was investigated by using the light-flash method. The thermophysical properties were affected by increasing the severity of the ageing conditions; thermal diffusivity decreased by up to 10% and specific heat by up to 5%.

## 1. Introduction

Solar energy is an essential source of renewable energy which provides a promising solution to the current climate crisis. One of the most mature of the solar technologies is concentrated solar power (CSP), which currently produces 0.5% of the global electricity and should provide 11% by 2050, according to References [1,2].

The main device employed in CSP technologies for exploiting solar energy is the solar collector, which is composed of a reflector and a receiver.

### State-of the-Art of Receiver Technology

Linear receivers and volumetric receivers are the two types of solar receivers currently available [3]. Linear receivers are mainly affected by thermal losses due to low thermal conductivity and large wall thickness. Volumetric solar receivers allow these shortcomings to be partially overcome. Volumetric receivers usually consist of a porous material that absorbs the concentrated solar radiation within the volume of a structure and transfers the absorbed heat to a fluid passing through the structure. Unlike linear receivers, volumetric receivers can exploit the “volumetric effect”, which allows the front side of the receiver to remain cooler than the working fluid.

The volumetric receiver technology has been under development since the early 1990s in various research and development projects [4]. The key element of the volumetric receiver is the absorber, which absorbs solar energy throughout its volume. High connected porosity and thermal conductivity are important requirements of the absorber material that all ow it to effectively transfer heat to the gases in the channels. Several materials have been examined for solar receivers [5]. Considering the high temperatures of over 800 °C reached in CSP plants, ceramic materials have so far proved to be the most suitable for absorbers [4,6]. 

The previous Phoebus-TSA Project [7,8] had focused on the application of metallic absorbers in volumetric receivers. In particular, the TSA initiative used wire-mesh metallic absorbers, with a limited air temperature of up to 550 °C, due to the high oxidation rates of the absorber material.

Then the SOLAIR Project (2000–2004) was developed to achieve higher operational temperatures by using ceramic absorbers [9,10,11,12]. In particular, in the SOLAIR (SOLar AIR) receiver, the wire-mesh was replaced with a SiC ceramic absorber characterized by an air-channeled structure. This improvement was aimed at increasing the air temperature up to 850 °C, to allow the receiver to work under higher solar radiation. Accordingly, SOLAIR had 30% less surface but achieved the same thermal power. Within the SOLAIR Project, the performances of 200 KW (SOLAIR 200) and 3 MW (SOLAIR 3000) receivers were analyzed. In SOLAIR 200, two of the three tested foam ceramic materials were able to withstand air temperature higher than 800 °C, with thermal efficiency of about 74 to 75%. In SOLAIR 3000, a SiC ceramic material characterized by 49.5% internal porosity was tested and achieved operational temperatures of 750 °C and radiation in the range of 370–520 KW/m^2^. However, sudden mechanical failure due to severe thermal cycling represented the limiting factor (up to 10–100 failures in first 500 h) in the SOLAIR Project [12]. 

The performance of solar receivers over time is adversely affected by three main factors [13]:

- The properties of the chosen material;
- The chosen cooling media, which affect the material corrosion and thermal stress;
- The operating conditions—high temperature and highly concentrated and abrupt solar flux—which can lead to high stresses during the lifetime.

The combination of these three factors is responsible for corrosion and fatigue mechanisms. Moreover, the degradation of the thermophysical properties of materials in laboratory conditions may be different from actual solar ageing. The differences between these two types of degradations (solar vs. thermal) are as yet not fully known [13].

Improving the efficiency of solar receivers by addressing failure from thermal cycling and thermal shocks is a primary driving factor in the European NEXTOWER research project (www.h2020-nextower.eu, accessed on 25 July 2021).

Porous SiC is therefore a promising material to be used in in next-generation solar receivers, due to its outstanding mechanical, thermal, and chemical robustness, which will be required for the receiver to operate under extreme thermal cycling without failure at maximum material temperatures of at least 800 °C and to deliver over 25 years of continued operation. The potential working lifetime of CSP plants might be simulated by subjecting the receiver materials to accelerated thermal ageing tests, with the aim of predicting the working lifetime of the receiver materials. Materials testing with accelerated thermal ageing can also provide data to design components with improved durability.

For the development of a new method, it is essential that the same type of ageing mechanisms occur in the materials during the accelerated testing as during normal in-service conditions. A general methodology that allows for all the factors associated with the work to be considered and a quantitative approach are necessary in order to be able to predict the expected service life of a component and the limitations in the service life set by the durability of its materials. [14]. Main previous test methods for solar furnaces and parabolic dishes are included in Table 1.

As far as the authors are aware, the research papers published so far have based their predictions of the receiver performances over time on mathematical models or have focused the thermophysical characterization after ageing on the assessment of a single specific thermal property, such as thermal diffusivity or specific heat.

Thermophysical characterizations are needed to identify the ageing conditions that most affect the properties of the samples, and to support new proposals for improvements to the current European and International Standards. A CEN (European Committee for Standardization) Workshop Agreement (CWA) (CWA 17726 High temperature accelerated ageing of advanced ceramic specimens for solar receivers and other applications under concentrated solar radiation, https://ftp.cencenelec.eu/EN/ResearchInnovation/CWA/CWA17726_2021e.pdf (accessed on 25 July 2021) has recently been published that defines the requirements, operation and analysis for very high temperature accelerated ageing of flat ceramic specimens for solar receivers under concentrated solar radiation. An informative Annex to ISO 18755 (ISO 18755:2005 Fine ceramics (advanced ceramics, advanced technical ceramics)—Determination of thermal diffusivity of monolithic ceramics by laser flash method https://www.iso.org/standard/31901.html (accessed on 25 July 2021) is currently being developed in order to allow the assessment not only of the thermal diffusivity, but also of the specific heat and thermal conductivity of ceramic materials by means of the laser/light flash method. The aim of the paper is to describe the effects of ageing on porous SiC samples, for which the thermophysical properties were evaluated. The study was based on accelerated ageing tests carried out on porous SiC large slab-shaped coupons (50 mm × 50 mm × 5 mm) in the CIEMAT-PSA solar furnace located in Almeria (Spain). In particular, non-destructive characterization by computed X-ray tomography and ultrasonic inspection was used to detect cracking and to enable the preparation of valid specimens for thermophysical characterization. The effects of thermal ageing on the thermophysical properties of the materials were assessed by comparing both the thermal diffusivity and the thermal conductivity of the aged and non-aged samples.

## 2. Materials and Methods

### 2.1. Material

Extrusion is the most common method for producing complex geometries of near-net shape, as well as planar samples [22,23], and for this study, slabs of porous SiC with dimensions of 50 × 50 × 5 mm were produced by extrusion and partially sintered by LiqTech Ceramics A/S (Ballerup, Denmark). Two different α-SiC powder batches were combined, with a particle size between 20 and 35 µm for the coarse batch and between 0.4 to 0.8 µm for the fine batch; this bi-modal granulometry of the SiC powders is necessary in order to achieve the necessary recrystallization mechanism during sintering. The powders were mixed together with a plasticizer (to give the necessary plasticity to the mass or paste), such as polyvinyl alcohol (PVA) [24]; a binder; a dispersant; and a mixture of water and ethanol as solvent. The extrusion slurry had a very high solid load, between 70 and 80 wt.%. solid. This slurry was stirred and extruded to produce the desired shape, with an extrusion pressure of no more than 30 bar, and the final shape was achieved by cutting after drying. The dried slabs were fired under argon atmosphere, at temperatures between 2100 and 2300 °C, for 1.5 h, in a graphite furnace, where the main sintering mechanisms were evaporation–condensation at the surface of the fine powders and diffusion at the contacts between the coarse particles [24,25]. To remove the residual carbon that may remain in the pores, a surface oxidation step was carried out at a temperature of 1100 °C, for 1 h, in an air furnace (ELS 1000 S SOB, Helmut Rohde GmbH, Prutting, Germany). The resulting slabs had a porosity around 43% and pore size of 17 µm [26].

### 2.2. Accelerated Ageing

In order to evaluate the thermal behavior of individual receiver ceramic components, an accelerated ageing test bench based on solar central receiver (SCR) technology was developed, installed, and tested at the CIEMA-PSA SF40 Solar Furnace (CIEMAT, Almeria, Spain) [27]. The test bench is composed of a 120 × 120 × 90 mm^3^ SiC honeycomb module, inside a 130 × 130 × 93 steel box that is open on its front side. Samples can be located on the honeycomb, on its front side, and opposite to the parabolic dish and may be heated by using concentrated solar energy directly on their exposed surface, similarly to a solar central receiver. A solar homogenizer or a compound parabolic concentrator (CPC) is placed in front of the samples in order to achieve a more homogenous solar flux on the samples. A main flux shutter controls the energy supply to the samples.

In accelerated tests, the cooling rate should be as fast as allowed by the material, within the test conditions and the limits of the sample, such as its maximum thermal gradient. Forced cooling is recommended for accelerating ageing, and a double forced air-cooling system was implemented in the test bench. In the thermal cycle, as soon as the working temperature is reached, the cooling part of the cycle starts. As the main flux shutter is closed and the forced cooling system is turned on, an inverted air blower that is connected to the back side of the steel box sucks the air around the samples and forces their cooling. A fast shutter closes to instantaneously block the solar flux on the samples.

**Figure 1:**
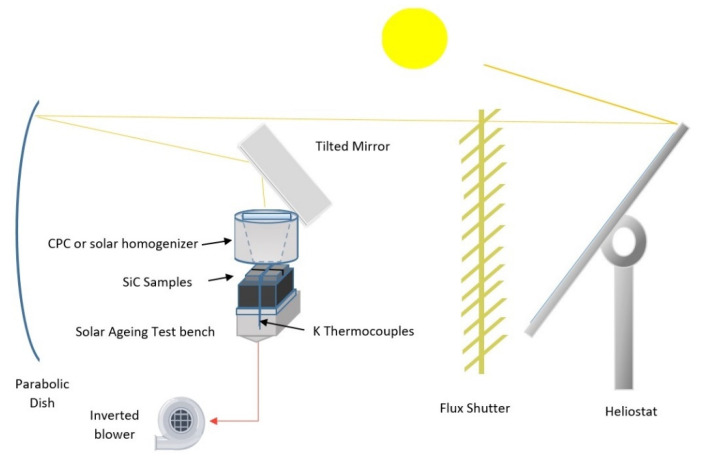
Schematic diagram of the solar ageing test bench in a horizontal solar furnace facility.

**Figure 2:**
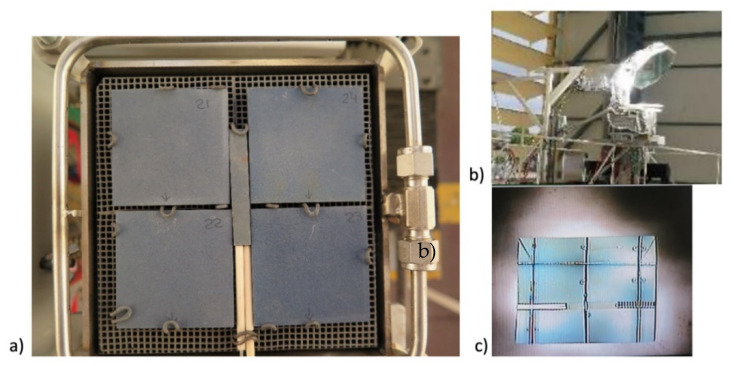
(**a**) Samples (50 mm × 50 mm × 5 mm) placed in horizontal position on the test bench before solar ageing testing. (**b**) Accelerated ageing test bench on test at SF40. (**c**) Slabs with thermocouples observed under concentrated solar radiation during solar ageing tests.

Figure 1 shows a schematic of the solar ageing test bench implemented at CIEMAT-PSA (Almeria, Spain). On the basis of preliminary ageing campaigns, the test bench was optimized by placing the samples on the horizontal plane in order to maximize the freedom of positioning and allow their thermal expansion. The cooling system, which is illustrated in Figure 2, allowed the thermal gradient inside the slabs to be better controlled. The goal is not to reach extreme conditions, but to conduct solar ageing in controlled conditions. A sacrificial SiC slab with 2 or 3 blind holes (K-type thermocouples inserted in each hole) is included in the setup in order to estimate the inner thermal gradient during the ageing cycles. The external part of each thermocouple is protected by a ceramic sleeve (Figure 2a). The control parameters are the maximum and minimum temperature and the thermal gradient in the sample, estimated by the thermocouple measurements and their relative position inside the sample. Shutter aperture and speed are used to control the thermal gradient in the sample.

This setup was used to carry out 500 ageing cycles at different periods, as described in Table 2, that were designed to cycle the inner temperature over a range from 300 up to 800 °C or 1000 °C, in order to compare the results with different temperatures and cycle periods. The cycle period (min/cycle) is the time needed to carry out a complete solar ageing cycle, including heating and cooling rate (Table 2). The maximum surface temperature (Tmax) and the flux (normalized at 1000 W/m^2^ isolation) are summarized in the same table, with values reaching up to 72 W/cm^2^. The maximum temperatures on the surface of the samples were estimated experimentally for each solar ageing cycle, taking into account the maximum temperature measured inside the sacrificial sample, the estimated thermal gradient obtained with the thermocouple measurement, and the relative position of the thermocouple in the sample. These experimental values are included in Table 2 as a range. The maximum temperature estimated for each group is the highest of its range. The cycle speed for the ageing tests up to 1000 °C was initially 10 min per cycle in order to achieve better control; the conditions were then optimized, thus allowing the cycle speed to be increased to 6 min per cycle (Table 2 and Table 3).

### 2.3. Post-Test Characterization

Slabs that had been exposed to accelerated ageing were examined by computed X-ray tomography (XCT), using a Zeiss Versa 510 X-ray microscope (22 µm/voxel resolution, 140 kV accelerating voltage, 10 W power, exposure of 14 s per projection with no binning, and 3001 projections per tomograph, Oberkochen, Germany). To reduce the scanning time, four samples exposed to the same thermal ageing conditions were examined simultaneously in a vertical stack with each sample in the horizontal plane. The tomographs were reconstructed by using the microscope software. For comparison, XCT examination of selected samples was also performed by using a Gilardoni high-energy system designed for industrial tomography (30 µm/voxel, 100 kV accelerating voltage, 28 W power, exposure of 2.5 s per projection with no binning, and 360 projections per tomograph, Mandello del Lario, Italy).

To verify the ability of XCT to detect cracking, a single slab of a similar (prototype) extruded SiC material (43% porosity, 50 × 50 × 5 mm) was observed by tomography at 20 µm/voxel (Zeiss Versa 510, 110 kV voltage, 10 W power, 6 s exposure time, 3001 projections over 360° rotation, and no binning, Oberkochen, Germany). The slab then received 10,000 cycles of thermal ageing (1 min/cycle) and was observed again, using the same microscope setup. Digital volume correlation (DVC) was then applied, using the LaVision DaVis software (sequential correlations with the minimum window size of 32 voxels, 75% overlap, Göttingen, Germany) to measure the relative 3D displacement fields. The correlation used the attenuation contrast from the internal porosity. An equivalent analysis of an identical slab, with a rigid body translation of 400 µm applied between tomographs that were obtained under the same conditions, demonstrated that DVC measured the local displacements with an uncertainty of 0.2 voxels (i.e., ~4 µm).

Ultrasound examination at 25 MHz with a Panametrics 5073PR system (Waltham, MA, USA) operating with immersion coupling at a focal length of 20 mm (6 mm probe diameter) was applied to selected slabs using an automated X-Y mapping system (step size 0.1 mm) developed at the ENEA, the National Agency for New Technologies, Energy and Sustainable Economic Development in Rome, Italy. This operates in the pulse-echo mode (Tektronics TDS 3032B, sampling rate 125 × 106 per second, Beaverton, Oregon, USA) to map the echo signal amplitude, which is dependent in the geometric size of defects. The three-dimensional amplitude matrix that is obtained can be represented by a point cloud for visualization.

Thermal diffusivity, α(T) (mm^2^/s), in the temperature range from 25 to 1000 °C was assessed through the Light Flash Analysis method (LFA) using a NETZSCH LFA 467 HT Hyperflash^®^ (Selb, Germany). This employs a Xenon lamp (Selb, Germany) to provide the flash, and an infrared detector measures the temperature rise of the sample as a function of time. The thermal diffusivity, α(T), was determined through the Parker method [28] that is based on the ratio between sample thickness and half-rise time (i.e., the time for the temperature of the rear surface of the sample to rise to half of the maximum temperature). The post-analysis was carried out through the improved Cape–Lehman approach [29,30] using the Proteus^®^ Software (Selb, Germany), thus allowing two-dimensional heat flows, heat losses and radiation effects above 500 °C to be assessed. The specific heat, $c_{p}\left(T\right),$ was evaluated indirectly by means of reference samples and the literature tables [31,32]. In particular, on the basis of specific heat values available in the literature [31], a reference sample was built for these analyses. Over the whole temperature range, the specific heat values were evaluated and assigned to a sample obtained from the first non-aged slab, which is the reference sample, as in Reference [32]. Thus, the thermal conductivity, *K(T)*, was obtained via Equation (1), using the sample’s density, ρ(T), which was determined by using the Archimedes method in deionized water.

(1) $$K\left(T\right)=\alpha \left(T\right)*c_{p}\left(T\right)*\rho \left(T\right)$$

## 3. Results

The experimental campaigns were conducted in order to compare the effect of different thermal-ageing conditions of the thermophysical properties of porous SiC samples. The test conditions were established by taking into account the realistic conditions of solar ageing, the possibilities provided by the facility and the temperatures of solar ageing campaigns that might be achieved in subsequent tests of SiC cups in a solar receiver. In particular, the cycles were conducted at three different speeds, in the temperature range between 300 and 800 °C, and at cycle speeds varying between 6 and 10 min/cycle in the temperature range between 300 and 1000 °C. The relevant thermal ageing parameters are listed in Table 2.

### 3.1. Microtomography Investigations

**Figure 3:**
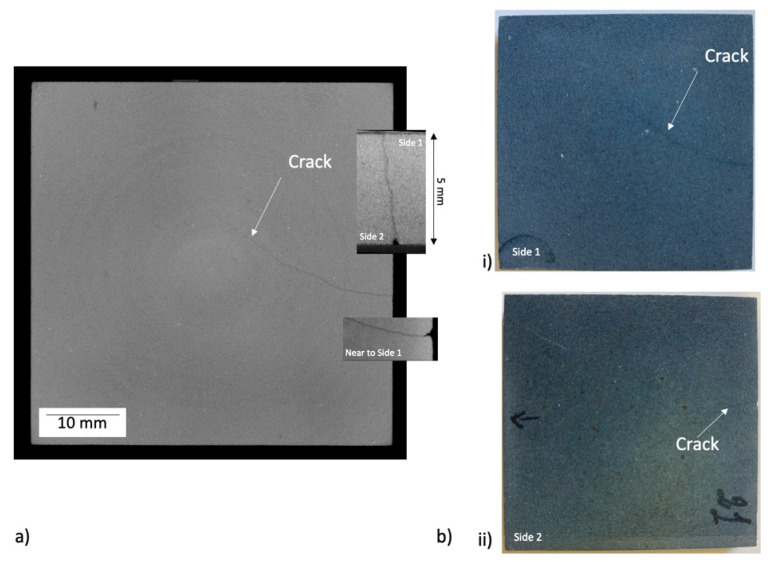
(**a**) µXCT cross-section of a slab (slab 21—see Table 2). A through thickness crack propagates from near the center of one edge, reaching the center of the slab. The “ring” contrast is an image artefact. (**b**) Optical images of the two surfaces of the slab. The crack is slightly visible on side 1 (unirradiated) and barely visible on side 2 (solar irradiated). All images show the full 50 mm × 50 mm slab.

**Figure 4:**
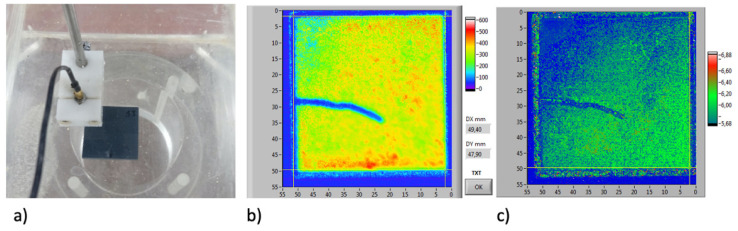
Ultrasonic inspection: (**a**) specimen (slab 23—see Table 2) undergoing ultrasonic mapping in pulse-echo mode, (**b**) amplitude map in C-scan model, and (**c**) amplitude map in D-scan mode. All images show the full 50 mm × 50 mm slab.

**Figure 5:**
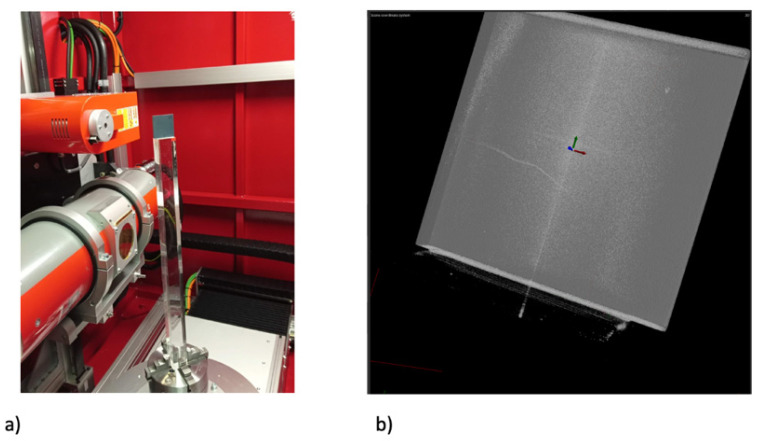
Industrial tomography (Gilardoni): (**a**) specimen (slab 23) within the tomography system; (**b**) 3D visualization within the full slab of crack, which propagates from the middle of the left edge towards the center. All images show the full 50 mm × 50 mm slab.

After performing the thermal ageing treatments, the slabs were observed by tomography to inspect for cracks. An example of a cracked specimen observed by high-resolution XCT (slab 21—see Table 2) is shown in Figure 3. The crack, clearly observed by XCT to be through the thickness, was barely visible by optical inspection (optical visibility was always better on the un-irradiated side, side 1, with no crack visible on the solar irradiated side, side 2, in most cases). Example data for a similar specimen (slab 23—see Table 1) undergoing ultrasonic inspection are shown in Figure 4a. Both the C-scan and D-scan (shown in Figure 4a,b, respectively) successfully detected the crack. The lower-resolution industrial tomography was also able to detect the crack successfully (Figure 5).

**Figure 6:**
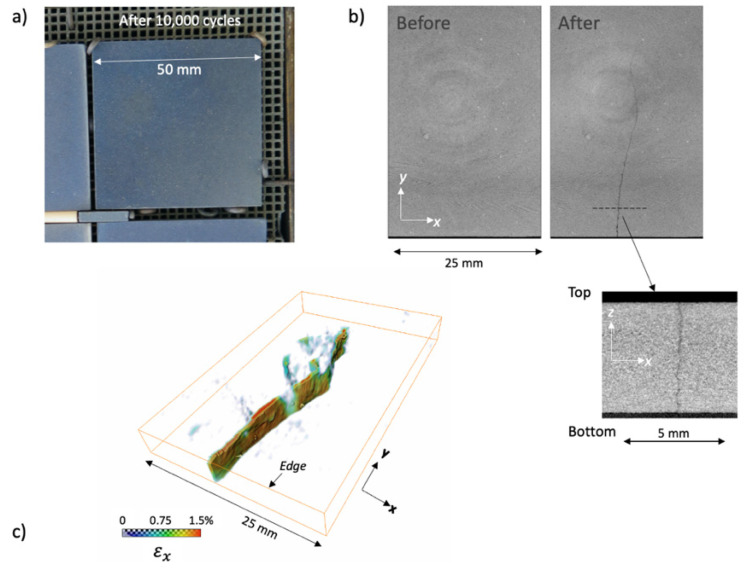
Characterization of solar aged prototype material: (**a**) optical image (in solar furnace) after 10,000 cycles; (**b**) XCT images (cropped) at the same midsection plane before and after solar ageing, with cross-section of the developed crack; (**c**) visualization of the crack, using the nominal strain in the x-direction, due to the relative displacements measured by digital volume correlation between tomographs.

Tomography found cracks in the four of specimens, specimens 21 to 24, which experienced the highest surface temperature of 1000 °C. Two of specimens 17–20 had detectable cracks (slabs 17 and 18; there were no cracks seen in slabs 19 and 20); of the specimens that were exposed to a maximum temperature of 800 °C; these four specimens were heated at the highest rate and experienced a higher thermal gradient during heating than those that were heated at a lower rate (Table 2). Only one crack was observed in each damaged specimen, and there were no cracks observed in slabs 8 to 16. In each case, the crack propagated towards the center of the specimen from near the center of one edge. The DVC analysis of high-resolution XCT images, when applied to the thermally cycled prototype material (Figure 6), showed that this cracked in a similar manner to the production quality material. The crack opening displacement can be visualized as a nominal strain, which describes the local gradient of the displacement field.

### 3.2. Thermophysical Characterizations

**Figure 7:**
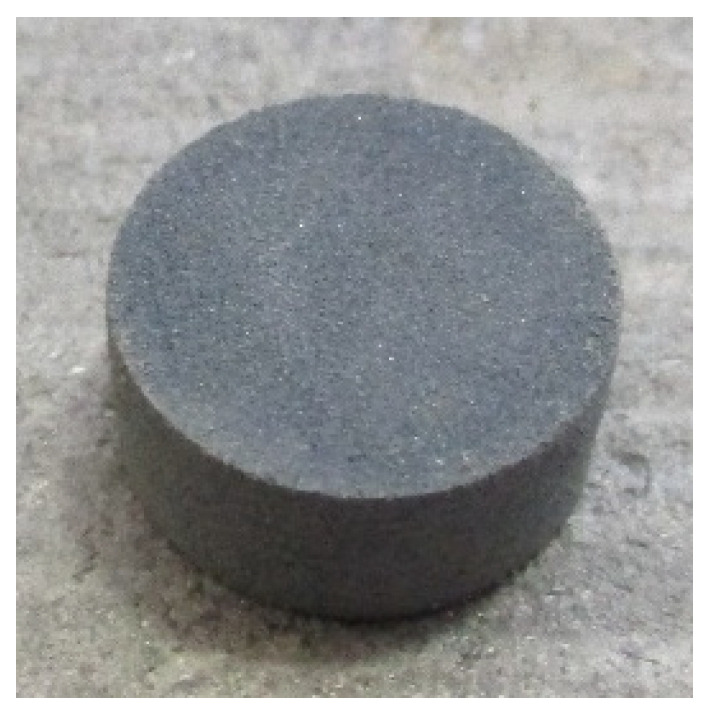
Example of the thermophysical sample (12.7 mm diameter and 5 mm thickness).

For the thermophysical characterization, circular samples were obtained by core drilling and subsequent machining from aged and non-aged slabs grouped according to Table 4. Figure 7 depicts an example of a sample. The tomography observations were used to avoid the detected cracks when preparing the thermophysical samples, which would otherwise be non-representative. The samples with the longest ageing cycles (5 min/cycle) were subjected to a lower temperature gradient than the samples subjected to the other thermal ageing conditions, and, significantly, the samples aged with slower cycles had no evident cracks. Two samples were analyzed for each group of slabs that represented the different ageing conditions. Table 4 summarizes the physical characteristics of the samples. Previous studies (see Reference [32] as an example) of the porous SiC analyzed in this paper found an average density of about 1800 kg/m^3^ and a deviation of ca. 2.8% (about 50 kg/m^3^) within the same production batch in the untreated condition. The variations of density for the individual samples in Table 4 are within this range and do not show an effect of ageing.

**Figure 8:**
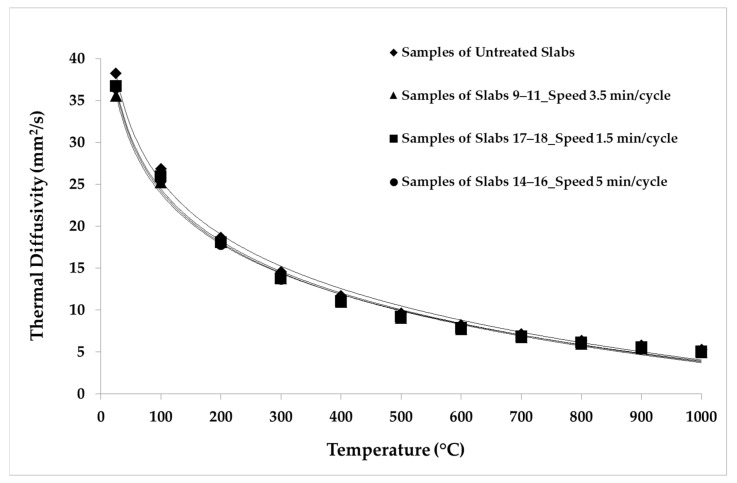
Thermal diffusivity values considering different speed of thermal cycles.

**Figure 9:**
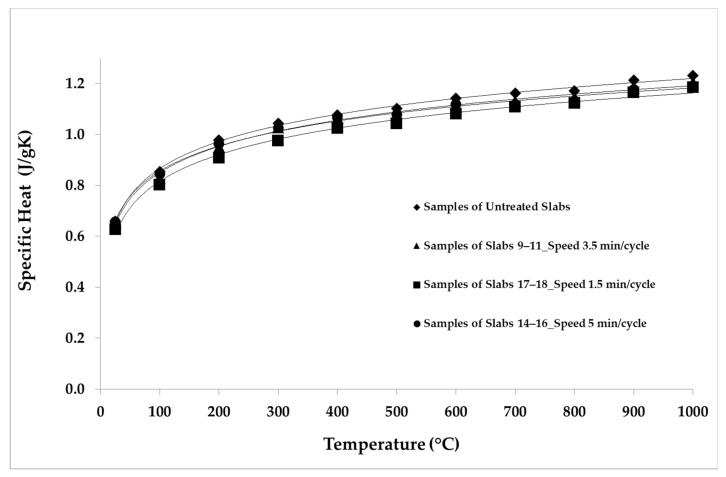
Specific heat values considering different speed of thermal cycles.

**Figure 10:**
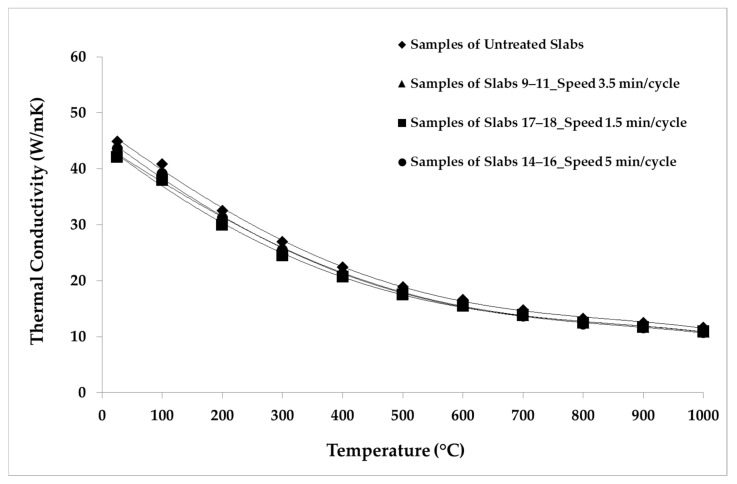
Thermal conductivity average values considering different speed of thermal cycles.

**Figure 11:**
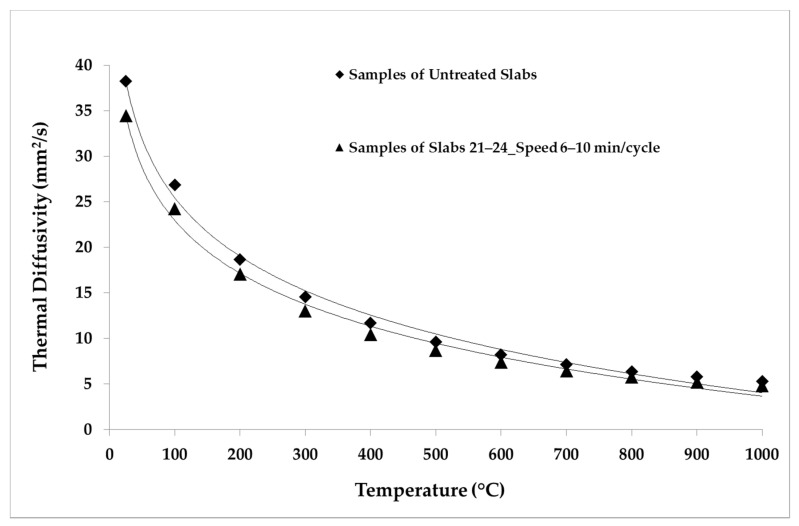
Thermal diffusivity values of untreated samples and samples aged in the temperature range 300–1000 °C.

**Figure 12:**
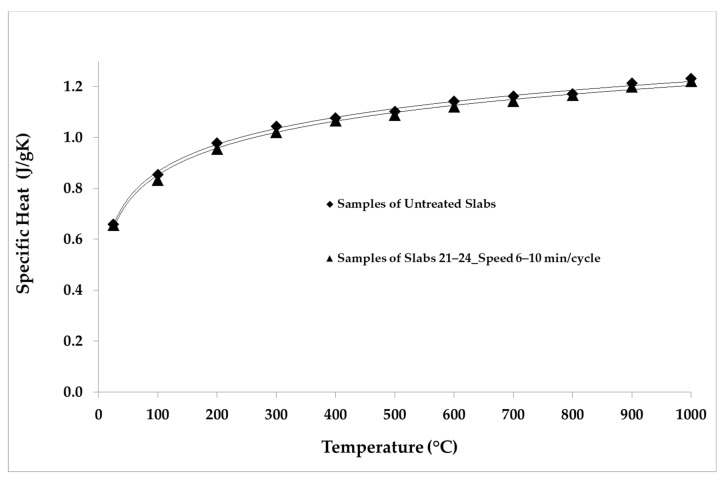
Specific heat values of untreated samples and samples aged in the temperature range 300–1000 °C.

**Figure 13:**
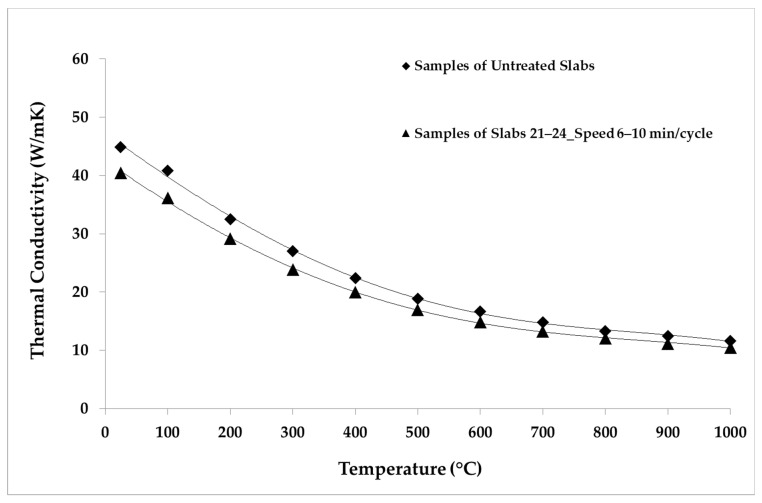
Thermal conductivity values of untreated samples and samples aged in the temperature range 300–1000 °C.

The LFA Method was employed for the thermal analysis of the samples. The parameters and the conditions of the analysis are shown in Table 5. The repeatability was verified by making five shots for each temperature point. The average values of the thermal properties measured for the samples belonging to each group of slabs are reported in Table 6, Table 7, Table 8, Table 9 and Table 10. The average values of the thermal properties of both the aged samples and the non-aged ones for each temperature point are also visualized in Figure 8, Figure 9, Figure 10, Figure 11, Figure 12 and Figure 13.

The specific heat of the samples increased with an increasing temperature (Figure 9). On the basis of specific heat values available in the literature [31], a reference sample was built for these analyses. Over the whole temperature range, the specific heat values were evaluated and assigned to a sample obtained from the first non-aged slab, which is the reference sample as given in Reference [32]. The specific heat of each of the other samples was assessed by comparing its temperature rise with that of the reference sample, as detailed in References [32,33]. The samples of groups 8–11 and 13–16, which were aged with longer cycles, showed a reduction in specific heat of about 2% compared to the non-aged samples, while the samples aged with shorter cycles presented an average reduction in specific heat of about 5%, which was particularly marked below 500 °C.

Figure 10 depicts the reduction in the thermal conductivity of the samples of groups 9–11 (3.5 min/cycle), 14–16 (5 min/cycle), and 17–18 (1.5 min/cycle) compared to the non-aged samples. The reduction was about 6% for the samples aged with longer cycles. The thermal conductivity of the samples aged with shorter cycles reduced by about 7% over the whole temperature range. The reduction in thermal conductivity is greater than the respective reductions in thermal diffusivity and specific heat, because conductivity is affected by both properties.

Figure 11, Figure 12 and Figure 13 show the comparison between the thermal diffusivity, specific heat and thermal conductivity of non-aged samples and of samples aged with longer cycles (speed 6–10 min/cycle), in the temperature range between 300 and 1000 °C. The thermophysical properties investigated were all reduced by about 10% over the whole temperature range with respect to the thermal properties of the non-aged samples, as shown below.

## 4. Discussion

The results show that the thermal ageing conditions of simulated solar cycles have an effect on the thermophysical properties of porous SiC slabs. Thermal diffusivity showed a decreasing trend with increasing temperature, due to the progressively higher phonon scattering [31,32]. For thermal cycles carried out in the same temperature range, i.e., between 300 and 800 °C, the thermal diffusivity of the aged samples decreased with the increasing speed of the ageing cycles. In particular, by comparing the samples of groups 9–11 (3.5 min/cycle), 14–16 (5 min/cycle), and 17–18 (1.5 min/cycle) with the non-aged (i.e., untreated) samples, it can be seen that thermal diffusivity reduced by about 6% in the whole temperature range for the samples with the longest ageing cycles, while diffusivity reduced by 5% for 3.5 min/cycle and by about 4% for 1.5 min/cycle. This confirms that the thermal diffusivity has been affected by thermal-ageing treatment.

By comparison between untreated and aged samples, it is also observed that, for samples aged over an inner temperature range from 300 to 800 °C, the specific heat was reduced by up to 5% in the samples aged with shorter cycles (Figure 9). This change is consistent with the literature findings, particularly those in Reference [34], where the specific heat of SiC and other materials was measured in order to understand the potential of each material to be used as a thermal energy storage medium in CSP plants; in that work, SiC showed a decrease in specific heat of about 8% after thermal ageing.

The observed change in thermal diffusivity and, hence, the thermal conductivity, is consistent with the literature findings of the effects of microcracking on thermal properties [35,36]. In particular, the study in Reference [36] shows that extensive microcracking in large-grain-sized materials (over 1 µm) significantly decreased thermal diffusivity. In this study, the mechanism for the decrease in thermal diffusivity is judged to be microcracking from thermal strains between the grains of the porous SiC. The mechanism for the observed reduction in specific heat after thermal ageing is not clear. Microcracking affects the porosity of the samples, but porosity has been shown to not significantly affect the specific heat, as reported in Reference [31], where the specific heat of porous SiC at different levels of porosity was determined.

The failure of porous materials due to very aggressive thermal ageing conditions has been reported in Reference [37]. In that work, it was concluded that the lack of formation of a protective oxide layer during ageing appeared to allow the fluxes to readily diffuse, due to the highly porous microstructure and the severe conditions of the thermal shock test. In this study, the shortest ageing cycles caused some slabs to crack. Samples tested over the larger temperature range of 300 to 1000 °C also cracked, despite the nominally lower thermal gradient. The cracks, which were detectable by non-destructive evaluation via X-ray tomography and ultrasound imaging, were optically visible on the back face of samples that was not irradiated by the solar flux. The fracture initiated near the middle of the sample edge and propagated towards the center. The initiation site is the expected location of maximum thermal stress in an unconstrained and simply supported rectangular slab that is heated uniformly on one planar surface [38]; hence, the thermal strains from higher thermal gradients encourage cracking. There is some inhomogeneity also of the surface temperature, as indicated in Figure 3, which would also contribute to thermal strains. It is also possible that cracking could be initiated if the thermal expansion of the slabs is restricted by the sample supports. Significantly, inspection by XCT or ultrasound allowed these cracks to be identified, and avoided, for the preparation of valid samples for the measurement of thermophysical properties.

This work has informed the CEN CWA *High temperature accelerated ageing of ceramic specimens and small solar receivers under concentrated solar radiation*, which describes the requirements that apply to the test pieces (shape and dimensions) and also to the test platform, in terms of suitability for supporting and holding the samples (position, freedom for thermal expansion, and compatibility between materials). It also includes guidance for the ageing procedure, with regard to the flux shape, the heating and cooling rates, the number of cycles and the temperature and incident flux measurement, and the analysis of tested samples. The thermophysical characterization conducted on these ceramic materials, which are to be employed in next-generation solar receivers, has laid the basis for the proposal of an informative Annex to ISO 18755, which is currently being developed in order to allow the complete characterization of ceramic materials by means of the flash method. The data from this thermophysical characterization may be used in numerical models to predict the thermal stresses in the cups that constitute the solar thermal receiver, in order to predict possible failure points. Accurate data for the bulk materials—in particular, their mechanical behavior—will enable prediction of the lifetime of the solar receivers.

## 5. Conclusions

Thermal ageing of porous (43% porosity) silicon carbide slabs with simulated solar cycles in a solar furnace was used to investigate the change in thermophysical properties. Macroscopic thermal strains caused fracturing in the slabs aged with shorter cycles and higher temperatures. These cracks were difficult to resolve visually, but could be identified reliably by using inspection by computed X-ray tomography and ultrasonic inspection, which allowed the preparation of valid specimens for the measurement of thermal properties. The thermophysical analyses showed a decrease in thermal diffusivity of up to 6%, with a greater decrease for shorter cycles over the same inner temperature range. This phenomenon may be explained by the development of micro-cracking, due to thermal strains between the grains of the porous SiC.

## Figures and Tables

**Table 1:** Selected test methods for materials or components tested in point focus facilities and resistively heat laboratory tube furnaces.

Material or Component Tested	Project/ Receiver	Facility	Conditions	Variables Measured	Reference
**RESISTIVELY HEAT LABORATORY TUBE FURNACES**					
Nickel Substrates for Coatings Plated from Four Different Bath Compositions	Sandia National Laboratories DE-AC04-76-DP00789.	Resistively heat laboratory tube furnaces - Sandia	Temperature range: 350 to 450 °C aging time: 100 h–5000 h.	Optical properties: solar absorptance and emittance	Pettit, R. B. 1983 [15]
SiC (Laboratory Prepared Materials)	Mirasol	Resistively tubular furnace	T: 1000 °C for 0 and 600 s. Cycles: 1, 2, 5 and 10 high temperature cycles	Reflectance values	Sallaberry, F. et al., 2015 [16]
**FRESNEL LENS**					
Metallic Samples (Iron Aluminides). Silicon Oxicarbides	Mirasol	Fresnel lenses: un-mounted in CENER, and on solar tracker in CSIC-CENIM	Heating rates: - Ambient to 800 °C: 50–70 °C/s. - 800 to 1000 °C: 3–5 °C/s. Cooling rates: - 1000 to 600 °C: 9–11 °C/s. - 600–400 °C: 2–4 °C/s - 400–200 °C: 1 °C/s.	Optical and mechanical properties, such as optical and scanning electron microscopy	Sallaberry, F. et al., 2015 [16]
**PARABOLIC DISHES**					
INCONEL 625	SFERA II	CIEMAT-PSA DISTAL	Aging temperature: 700 °C Period: up to 500 h. Samples: 5 tubular samples	Evolution of microstructure and mechanical properties: SEM, optical microscopy. Vickers hardness	Setien, E.; et al., 2017 [17]
**SOLAR FURNACES**					
SIRCON foam absorber made of SIRCON (Si_3_N_4_)	PLVCR-5	Sandia (SNLA) Solar furnace	T average outlet air: 625 °C T max outlet air: up to 1000 °C Power: 5 kW P 10 bar Flux_max level: 2 MW/m^2^	Testing in solar conditions	Pritzkow, W. 1991 [18]
INCONEL with Pyromak 2500 layer	PROMES-CNRS	PROMES-CNRS SAAF	Samples: 1 mm Inconel (after spray-gun application of paint coating). Mean irradiance: 104 kW/m^2^–173 kW/m^2^, 346 kW/m^2^ Period: 10 s, 30 s, Exposure time: 1000 s, 3000 s	Normal solar absorptance, thermal effusivity Thermal conductivity, thermal contact resistance between coating and substrate.	Boubault A., et al., 2014 [19]
Ceramic foams (SiC and ZrB_2_) as high temperature volumetric solar absorber	OPTISOL project, SFERA project, and STAGE-STE	PROMES-CNRS 6 kW solar furnace. Kaleidoscope solar flux homogenizer	Tout: 833 to 998 °C Mass flow rate: 1 g/s. α-SiC, Si-SiC, SiC, SiC + Al_2_O_3_, SiC + SiO_2_ + Al_2_O_3_, ZrB_2_. SiC range of porosity (72–92%)	Calorimetry and fluxmetry	Mey-Cloutier, S. et al., 2016 [20]
Mullite	STAGE-STE. SFERA II H2CORK project	CIEMAT-PSA SF40	Tmax: average value of 1180 ± 35 °C T differences ranging - 200 °C (700–900 °C); - 400 °C (700–1100 °C) - 600 °C (700–1300 °C)	Mechanical properties: Typical strength versus strain microscopic techniques (SEM and optical microscopy).	Oliveira, F.A.C., et al., 2019 [21]

**Table 2:** Parameters of the experimental ageing test conditions.

Group of Slabs	Number of Cycles	Inner Temperature Range (°C)	Periods (min/Cycle)	Thermal Gradient Max (°C/mm)	Tmax Surface (°C)	Flux -Normalized at 1000 W/m^2^ Insolation (W/cm^2^)
Slabs 17, 18, 19, 20	500	300–800	1.5	≅20–24	860–920	69–72
Slabs 8, 9 10, 11	500	300–800	3.5	≅22	850–900	40–42
Slabs 13, 14, 15, 16	500	300–800	5	≅10–13	840–880	42–44
Slabs 21, 22, 23, 24	500	300–1000	6–10	≅10–14	1000–1050	47–55

**Table 3:** Average values of heating and cooling rates during the solar ageing tests.

Group of Slabs	Number of Cycles	Inner T Range (°C)	Periods (min/Cycle)	Heating Rate Average Values (°C/s)	Cooling Rate Average Values (°C/s)	Max Heating Rate (°C/s)	Max Cooling Rate (°C/s)
Slabs 17, 18, 19, 20	500	300–800	1.5	22.5	7.0	50	30
Slabs 8, 9 10, 11	500	300–800	3.5	6.5	3.0	20	16
Slabs 13, 14, 15, 16	500	300–800	5.0	5.0	2.5	16	11
Slabs 21, 22, 23, 24	500	300–1000	6.0	4.0	5.0	10	30
			10.0	2.0	3.0	6	30

**Table 4:** Physical characteristics of the samples (all diameter 12.7 mm).

Group of Slabs	Sample	Thickness (mm)	Density (kg/m^3^)
Untreated slabs	Sample from slab 1	5.808	1780
	Sample from slab 2	5.714	1783
Group 8–11	Sample 1 from slab 9	5.648	1849
	Sample 2 from slab 11	6.014	1788
Group 13–16	Sample 1 from slab 14	5.662	1836
	Sample 2 from slab 16	5.728	1809
Group 17–20	Sample 1 from slab 17	5.707	1836
	Sample 2 from slab 18	5.674	1817
Group 21–24	Sample 1 from slab 21	5.774	1777
	Sample 2 from slab 24	5.570	1819

**Table 5:** Parameters and conditions of the thermophysical characterization carried out through the Light Flash Apparatus.

Temperature Range (°C)	Temperature Steps (°C)	Inert Atmosphere	Heating Rate (K/min)
25–1000 °C	100-(11 experimental points)	Argon	4

**Table 6:** Thermophysical properties of the untreated samples. Average values of the two samples.

Temperature (°C)	Thermal Diffusivity (mm^2^/s)	Specific Heat (J/gK)	Thermal Conductivity (W/mK)
25	38.226	0.660	44.895
100	26.861	0.854	40.843
200	18.659	0.979	32.532
300	14.528	1.044	26.998
400	11.692	1.077	22.416
500	9.597	1.103	18.853
600	8.188	1.142	16.659
700	7.148	1.162	14.795
800	6.357	1.173	13.275
900	5.775	1.214	12.481
1000	5.293	1.232	11.613

**Table 7:** Thermophysical properties of the aged samples obtained from slabs 9 and 11. Average values of the two samples.

Temperature (°C)	Thermal Diffusivity (mm^2^/s)	Specific Heat (J/gK)	Thermal Conductivity (W/mK)
25	35.600	0.655	42.454
100	25.227	0.829	38.050
200	18.125	0.952	31.359
300	13.773	1.028	25.724
400	11.007	1.064	21.294
500	9.100	1.080	17.878
600	7.749	1.113	15.682
700	6.769	1.131	13.925
800	6.014	1.147	12.549
900	5.456	1.184	11.744
1000	4.993	1.205	10.944

**Table 8:** Thermophysical properties of the aged samples obtained from slabs 14 and 16. Average values of the two samples.

Temperature (°C)	Thermal Diffusivity (mm^2^/s)	Specific Heat (J/gK)	Thermal Conductivity (W/mK)
25	36.478	0.657	43.605
100	25.476	0.846	39.191
200	17.809	0.963	31.239
300	13.663	1.026	25.508
400	10.913	1.068	21.240
500	9.036	1.078	17.746
600	7.703	1.119	15.707
700	6.719	1.118	13.690
800	5.967	1.127	12.258
900	5.407	1.177	11.592
1000	4.962	1.188	10.742

**Table 9:** Thermophysical properties of the aged samples obtained from slabs 17 and 18. Average values of the two samples.

Temperature (°C)	Thermal Diffusivity (mm^2^/s)	Specific Heat (J/gK)	Thermal Conductivity (W/mK)
25	36.724	0.627	42.093
100	25.898	0.802	37.944
200	18.059	0.908	29.959
300	13.755	0.975	24.496
400	11.053	1.024	20.679
500	9.194	1.043	17.512
600	7.845	1.082	15.499
700	6.843	1.108	13.851
800	6.084	1.124	12.488
900	5.516	1.165	11.735
1000	5.047	1.186	10.928

**Table 10:** Thermophysical properties of the aged samples obtained from slabs 21 and 24. Average values of the two samples.

Temperature (°C)	Thermal Diffusivity (mm^2^/s)	Specific Heat (J/gK)	Thermal Conductivity (W/mK)
25	34.426	0.656	40.427
100	24.245	0.833	36.156
200	17.032	0.956	29.154
300	13.032	1.021	23.856
400	10.438	1.066	19.952
500	8.672	1.088	16.919
600	7.408	1.121	14.893
700	6.461	1.143	13.243
800	5.754	1.166	12.035
900	5.197	1.199	11.178
1000	4.795	1.221	10.500

## Data Availability

The data presented in this study are available upon request from the corresponding author. The data are not publicly available, due to the use of proprietary manufacturing processes.
